# Antioxidant potential of alkaloids and polyphenols of *Viola canescens* wall using *in vitro* and *in silico* approaches

**DOI:** 10.3389/fchem.2024.1379463

**Published:** 2024-04-12

**Authors:** Imtiaz Ahmad, Pin-Jui Huang, Nosheen Malak, Adil Khan, Fayaz Asad, Chien-Chin Chen

**Affiliations:** ^1^ Department of Botany, Bacha Khan University Charsadda, Charsadda, Pakistan; ^2^ Division of Surgical Intensive Care Unit, Department of Surgery, Ditmanson Medical Foundation Chia-Yi Christian Hospital, Chia-Yi, Taiwan; ^3^ Department of Zoology, Abdul Wali Khan University, Mardan, Pakistan; ^4^ Department of Pathology, Ditmanson Medical Foundation Chia-Yi Christian Hospital, Chiayi, Taiwan; ^5^ Department of Cosmetic Science, Chia Nan University of Pharmacy and Science, Tainan, Taiwan; ^6^ Doctoral Program in Translational Medicine, Rong Hsing Translational Medicine Research Center, National Chung Hsing University, Taichung, Taiwan; ^7^ Ph.D. Program in Translational Medicine, Rong Hsing Research Center for Translational Medicine, National Chung Hsing University, Taichung, Taiwan; ^8^ Department of Biotechnology and Bioindustry Sciences, College of Bioscience and Biotechnology, National Cheng Kung University, Tainan, Taiwan

**Keywords:** antioxidants, *Viola canescencs*, flavonoids, *in silico*, *in vitro*

## Abstract

**Background:** *V. canescens* Wall, a plant renowned for its ethno-medical properties, was investigated in this study for its antioxidant potential based on its wide therapeutic applications in traditional healthcare systems. The study aimed to assess the antioxidant potential of the plant extract/fractions and to predict the active phytochemicals using computational techniques.

**Methods:** Five fractions were obtained from the crude methanolic extract of *Viola canescens,* and six concentrations (25, 50, 75, 100, 125, and 150 μg/mL) were prepared for each fraction. The antioxidant activity of these fractions was evaluated using the Tetraoxomolybdate (VI) and 1,1-diphenyl-2-picrylhydrazyl (DPPH) assay. *In-silico* docking studies and molecular dynamic simulations were conducted to further elucidate the molecular interactions underlying the antioxidant activity.

**Results:** The aqueous extract of *V. canescens* exhibited significant antioxidant and free radical scavenging activity against DPPH. Additionally, the crude flavonoid extract demonstrated moderate activity with IC_50_ value of 57.863 μg/mL, indicating potent inhibition of cell growth. *In-silico* docking studies revealed a strong interaction between emetine and the aromatase protein, suggesting its potential as an antioxidant.

**Conclusion:** The study findings highlight the antioxidant potential of *V. canescens* extract, indicating its suitability as a source of natural antioxidants. These results suggest its potential application in pharmaceutical preparations aimed at harnessing antioxidant properties for therapeutic purposes.

## 1 Introduction

*Viola canescens* wall belongs to the family Violaceae and is a small herb (10–25 cm tall), having immense ethnomedicinal importance. In traditional healthcare systems, the plant is utilized for several therapeutic purposes, including carminative, antipyretic, anti-inflammatory, and antinociceptive agents. The whole plant is also used to treat cancer and numerous nerve disorders. Decoction of the plant is used as an aphrodisiac agent, relieving asthma and headache, and as an antiseptic agent (Muhammad and Dirk, 2010; Ahmad et al., 2011).

Free radicals are chemical species possessing unpaired electrons. Oxidants are free radicals possessing unpaired electrons and have strong affinities with oxygen and nitrogenous species. Free radicals are produced in the body as a byproduct of various metabolic pathways. These radicals alter the normal functioning of cells by altering the configuration of major biomolecules like proteins, lipids, and nucleic acids, causing cell damage that leads to severe health issues including but not limited to cancer, diabetes, neurological disorders, and decreased immunity and increased aging symptoms (Kalita et al., 2013). Living organisms have inherited mechanisms to neutralize and counter the adverse effects of free radicals. The ability of living organisms to eliminate free radicals is negatively affected by various factors, predominantly aging (De Torre et al., 2019). To avoid the harmful impacts of free radicals, organisms need antioxidants that are either produced endogenously by organisms or provided exogenously. Important endogenous antioxidants include catalase, peroxidase, and vitamin A (Sajjadi et al., 2019). Typically, the exogenous antioxidants work to boost the inherited antioxidant-eliminating mechanisms of living organisms (Göçer and Gülçin, 2011).

Several plants have been identified to exhibit antioxidant potential associated with their ability to produce a wide range of phytochemicals, most importantly polyphenols and alkaloids (Zengin et al., 2010; Tavassoli and Djomeh, 2011). In the current study, different fractions of the whole plant of *V*. *canescens* were screened for their antioxidant potential based on the traditional therapeutical application of the plant and the presence of important phytochemicals like alkaloids (Emetine), flavones (Vioanthin), and flavonoids (quercetin).

## 2 Materials and methods

### 2.1 Plant collection

Whole plants of *V. canescens* were collected at the flowering stage from April to June (2014–2015) from different localities of District Swat and Dir. Plant identification was made using standard literature (Qasier and Omer, 1985) and further confirmed through the Department of Botany, University of Peshawar. The identified specimen was kept at the Herbarium, University of Peshawar, for the record.

### 2.2 Preparation of extracts and fractions

Plant material was soaked in methanol at a rate of 100 g of powder per 250 mL of solvent in a closed container for 7 days. During the soaking time, the mixture was vigorously shaken at regular intervals. The mixture was then filtered using Whatman filter paper No. 1. The filtrate was dried using a rotary evaporator to obtain crude methanolic extract (CME). Organic solvent fractions (n-Hexane (NHF), ethyl acetate (EAF), chloroform (ChF), and aqueous fraction (AqF) of methanolic extracts were prepared using separating funnels. Furthermore, standard protocols separated crude alkaloids (CTA) and crude flavonoids (CTF) from methanolic extracts.

### 2.3 Dose exposure conditions

To ensure a comprehensive evaluation of the antioxidant potential of *V. canescens* extracts and fractions, a systematic dose-response approach was adopted. Five fractions were obtained from the methanolic crude extract of powdered plant parts: N-Hexane Fraction (NHF), Ethyl Acetate Fraction (EAF), chloroform Fraction (ChF), Aqueous Fraction (AqF), and Crude Total Alkaloid (CTA). Additionally, a Crude Total Flavonoid (CTF) extract was prepared. Each fraction was subjected to six concentrations (25, 50, 75, 100, 125, and 150 μg/mL) to cover a wide range of doses. These concentrations were selected to facilitate the observation of dose-response relationships and to ensure comprehensive evaluation of antioxidant potential.

### 2.4 Determination of total antioxidant potential

The total antioxidant potential of plant extracts/fractions was assessed following the standard protocol (Prieto et al., 1999) with minor modifications. In the assay, the antioxidant capacity of the samples (extract/fraction) is determined based on their ability to reduce Tetraoxomolybdate (VI) to pentaoxomolybdate (VI). Six concentrations, i.e., 25, 50, 75, 100, 125, and 150 μg/mL of crude methanolic extract (CME), N-Hexane Fraction (NHF), Ethyl Acetate Fraction (EAF), chloroform Fraction (ChF), Aqueous Fraction (AqF), and Crude Total Alkaloid (CTA) and Crude Total Flavonoid (CTF) were prepared. 0.5 mL of each concentration of extract/fractions were mixed separately with a 3 mL reagent mixture comprised of H₂SO₄ (0.6 M), Na₃PO₄ (28 mM), and (NH4)_2_MoO_4_ (1%) in test tubes. All the test tubes containing the mixture of extract/fractions and reagent were incubated for 10 min at 95°C. Thereafter, the mixtures were cooled to room temperature, and absorbance was recorded at 695 nm using a double-beam spectrophotometer. Ascorbic acid was selected as a positive control, while a blank solvent with 3 mL of reagent mixture was taken as a negative control. % Total antioxidant potential and IC_50_ were calculated for each extract/fraction.

$$\%\text{ Total antioxidant potential}=\frac{Absorbance\;in\;control-absorbance\;in\;sample}{Absorbance\;in\;control}\times 100$$

### 2.5 DPPH radical scavenging assay

The DPPH radical scavenging potential of extracts/fractions was evaluated using the standard protocol following (Saeed et al., 2012). Selected dilutions (20 μg/mL, 40 μg/mL, 60 μg/mL, 80 μg/mL, and 100 μg/mL) of CME, NHF, EAF, ChF, AqF, CTA and CTF were prepared. 5mL of each dilution was mixed separately with 1 mL of 0.001 M of DPPH in separate test tubes and placed in complete darkness for 1 h to complete the reaction. Ascorbic acid was used as positive control in the assay, while a mixture of DPPH and blank solvent was treated as negative control. After 1 hour of incubation, absorbance at 517 nm for each mixture was recorded using a double-beam spectrophotometer.

$$\%\;Inhibition=100-\frac{Absorbacne\;in\;control-Absorbance\;in\;test}{Absorbnce\;in\;control}\times 100$$

### 2.6 Docking studies

#### 2.6.1 Selecting the appropriate receptors and ligands

The three phytochemicals that were taken into account for the investigation were produced by *V. canescens* plants. In a review of the literature, the targets for breast cancer were determined to be the progesterone receptor (PR) and the aromatase protein. The X-ray crystal structures of the PR protein and co-crystallized ligand (PDB ID: 4OAR) and the aromatase protein and co-crystallized ligand were made available by Protein Data Bank (PDB ID: 3S7S).

#### 2.6.2 Preparation of the ligand

Chem’s 2D and 3D choices were used to depict each of the substances that were selected. It was done by saving a mol2 file with Chem Draw software (Mendelsohn, 2004). Auto dock tools (version 1.5.6) were utilized to get atomic coordinates after these molecules were converted to Pdbqt file format. Energy reduction, conformational analysis, and ligand generation were done with the help of the BIOVIA Discovery Studio application.

#### 2.6.3 Target active binding sites analysis

Using Drug Discovery Studio version 3.0, the target protein’s active binding sites were investigated. The ligand’s positions in the first target protein grids correspond to the active sites.

#### 2.6.4 Molecular docking studies

PR and aromatase were chosen as targets for breast cancer based on the literature. Protein Data Bank was used to get the X-ray crystal structures of PR and co-crystallized ligand (PDB ID: 4OAR) and Aromatase and co-crystallized ligand (PDB ID: 4DRH). The Auto Dock Vina software was loaded with the potential binding configurations between the ligands and the target proteins 4OAR and 3S7S. Using Autodock Tools (version 1.5.6), protein structures were generated as appropriate docking targets. The protein structures were cleaned of water molecules, metal atoms, co-crystallized ligands, and other noncovalently bound molecules. After adding the gesteiger charges, the target file was saved in the appropriate pdbqt format. When the structure is saved, the software automatically adds polar hydrogen and merges nonpolar hydrogens. Utilizing the 2D and 3D options in Chem Draw, ligand structures for three chemicals from the *V. canescens* plant were created and saved in mol2. Software called Autodock Vina (http://vina.scripps.edu/download.html) was used to analyze docking (Trott and Olson, 2010). Re-docking the native ligands into their original binding pockets served as proof of the effectiveness of our suggested docking technique. The experimental interaction conformation was superimposed on the anticipated docked conformation, and the root means square deviation values for the two poses were computed. Only protein structures with RMSD values between the native ligand’s docked and experimental poses below a 2 Å threshold were subjected to docking investigations. To best suit the active binding site, the grid box corresponding to the docking search space was modified. The DG binding energy values (kcal/mol) for the docked ligand structures were recorded as the results. Using Accelerys Discovery Studio 4.1 (Dassault Systems Biovia, San Diego, CA, United States), interactions between proteins and ligands were explored.

#### 2.6.5 Properties of drug-likeness

By applying Lipinski’s rule of five and using Molinspiration’s (http://www.molinspiration.com) molecular properties and bioactivity prediction, the compounds were examined for drug-likeness. Hydrogen bond acceptors (not more than 10), hydrogen donors (not more than 5), partition coefficient (not more than 5), rotatable bonds (less than 10), total polar surface area (not more than 140), and molecular weight (less than 500 g/mol) were used to assess the drug-likeness. The compounds’ SMILES formats were downloaded from the PubChem database at https://pubchem.ncbi.nih.gov/.

#### 2.6.6 Molecular dynamic simulation

iMODs server was used to simulate molecular dynamics to evaluate the stability and motion of the docked complex of the target protein for Emetine. Using normal mode analysis (NMA) to compute the internal coordinates, the iMOD service assesses the stability of proteins. The stability of the protein was shown using the eigenvalue, covariance matrix, main-chain deformability plot, elastic network model, and B-factor values (López-Blanco et al., 2014).

**Ethical Approval:** The was approved by the Ethical Committee of Biological Sciences Section, Department of Botany, University of Peshawar, Pakistan, under approval no. 99-J-10849.

## 3 Results

### 3.1 Determination of total antioxidant potential

Extracts/fractions of *V. canescens* used in the assay exhibited significant antioxidant potential in comparison to negative control. All selected extracts/fractions showed optimum potential at the highest concentrations, indicating a strong positive correlation between antioxidant potential and dose concentrations. Maximum potential was recorded for CTF (IC_50_ = 57.863), followed by EAF (IC_50_ = 65.066) and AqF (IC_50_ = 68.557), most probably due to the presence of polyphenols in these fractions. CTA and CME also exhibited promising potentials with IC_50_ values of 78.809 and 72.066, respectively. NHF was found to be the least effective treatment in the assay with IC_50_ = 123.499 (Table 1).

**TABLE 1:** Total antioxidant potential of selected extract/fractions of *Viola canescens*.

Concentrations	Treatments						
	CME	EAF	AqF	NHF	ChF	CTA	CTF
	Total antioxidant potential						
25 μg/mL	40.23%	40.23%	40.03%	33.72%	40.61%	38.31%	42.91%
50 μg/mL	41.09%	47.64%	45.45%	39.64%	41.45%	41.09%	48.73%
75 μg/mL	50.52%	51.92%	50.17%	42.16%	50.17%	45.30%	51.92%
100 μg/mL	51.27%	55.38%	54.0%	49.05%	50.95%	53.25%	55.38%
125 μg/mL	58.26%	56.7%	57.63%	51.09%	57.94%	57.63%	58.26%
150 μg/mL	63.47%	60.48%	60.48%	52.69%	64.37%	62.28%	63.17%
IC50	72.066	65.185	68.557	123.499	73.006	78.809	57.863

**CME**: crude methanolic extract from methanolic extracts; **EAF**: ethyl acetate fraction; **AqF**: aqueous fraction; **NHF**: Organic solvent fractions (n-Hexane fraction).

### 3.2 DPPH radical scavenging assay

In the DPPH radical scavenging assay, the antioxidant potential is assessed by recording the absorbance of a mixture of DPPH and extract/fraction at 517 nm. For DPPH stable radicals, the maximum absorbance occurs at 517 nm (Kedare and Singh, 2011). The extract/fraction possessing antioxidant potential lowers the absorbance by converting DPPH to DPPH-H through the ability of H+ ions to donate (Huang et al., 2005). Extracts/fractions of *V. canescens* used in the assay significantly reduced the absorbance at 517 nm. Furthermore, the maximum decrease in absorbance for extracts/fractions was recorded at the highest concentration (100 μg/mL). At 100 μg/mL concentration, absorbance for CME, EAF, AqF, NHF, ChF, CTA, and CTF was recorded as 0.21 ± 0.002, 0.30 ± 0.003, 0.30 ± 0.003, 0.26 ± 0.003, 0.20 ± 0.001, 0.18 ± 0.003 and 0.21 ± 0.004 respectively (Table 2). At highest concentration (100 μg/mL), maximum DPPH radical scavenging activity (70.28%), with IC_50_ value of 24.23 was recorded for CTA, followed by CTF, ChF with IC_50_ values of 26.71 and 60.45 respectively (Table 3). Overall percent radical scavenging activities for the used extract/fraction was in the following order.

**TABLE 2:** Absorbance of selected extract/fractions of *Viola canescens* in DPPH radical scavenging assay.

Treatments	Absorbance at 517 nm				
	20 μg/mL	40 μg/mL	60 μg/mL	80 μg/mL	100 μg/mL
CME	0.45 ± 0.001^a^	0.43 ± 0.001^a^	0.36 ± 0.003^a^	0.33 ± 0.003^a^	0.21 ± 0.002^a^
EAF	0.45 ± 0.001^a^	0.40 ± 0.004^a^	0.36 ± 0.002^a^	0.34 ± 0.004^a^	0.30 ± 0.003^a^
AqF	0.48 ± 0.007^a^	0.35 ± 0.004^a^	0.34 ± 0.001^a^	0.32 ± 0.002^a^	0.30 ± 0.002^a^
NHF	0.40 ± 0.002^a^	0.37 ± 0.003^a^	0.33 ± 0.004^a^	0.30 ± 0.001^a^	0.26 ± 0.003^a^
ChF	0.44 ± 0.039^a^	0.36 ± 0.005^a^	0.33 ± 0.003^a^	0.32 ± 0.003^a^	0.20 ± 0.001^a^
CTA	0.22 ± 0.003^a^	0.22 ± 0.002^a^	0.21 ± 0.002^a^	0.18 ± 0.001	0.18 ± 0.003^a^
CTF	0.32 ± 0.002^a^	0.28 ± 0.002^a^	0.27 ± 0.004^a^	0.21 ± 0.003^a^	0.20 ± 0.004^a^

^a^ : significantly different from control (negative) at α < 0.001; **IC**_**50**:_ Concentration causing 50% scavenging activity.

**TABLE 3:** Percent DPPH scavenging potential and IC50 values of selected extract/fractions of *Viola canescens*.

Treatments	% DPPH scavenging activity					
	20 μg/mL	40 μg/mL	60 μg/mL	80 μg/mL	100 μg/mL	IC_50_
CME	28.91	33.51	43.41	50.21	65.35	72.45
EAF	29.31	37.52	43.43	47.45	50.13	88.23
AqF	21.28	42.31	46.03	50.34	53.03	79.31
NHF	37.69	42.02	49.08	54.41	59.06	63.32
ChF	32.89	43.04	49.25	49.94	66.09	60.45
CTA	64.21	66.31	67.05	68.47	70.28	24.23
CTF	47.31	53.51	57.31	65.51	67.29	26.71

CTA > CTF > ChF > NHF > CME > AqF > EAF.

### 3.3 Molecular docking analysis’s *in silico* prediction of an antioxidant mechanism based on protein targets

The discovery of new drugs frequently uses plant secondary metabolites and phytocompounds as natural starting molecules. Their biological features result from their capacity to either target or control the activity of important enzymes involved in oxidative damage, inflammation, or cell proliferation (Cox-Georgian et al., 2019). The ability to forecast such potential biological impacts is continuously improving due to state-of-the-art computer approaches. These computational techniques are widely applied at various stages of contemporary drug discovery research, assisting researchers in their continual search for highly effective medicinal active substances. A valuable method that can provide a deeper comprehension of the possible mechanisms by which *in vitro* biologically active compounds function is molecular docking. Here, we employed molecular docking to locate a putative additional protein-targeted mode of action associated with the potential antioxidant effect of *V. canescens in vitro*.

**FIGURE 1:**
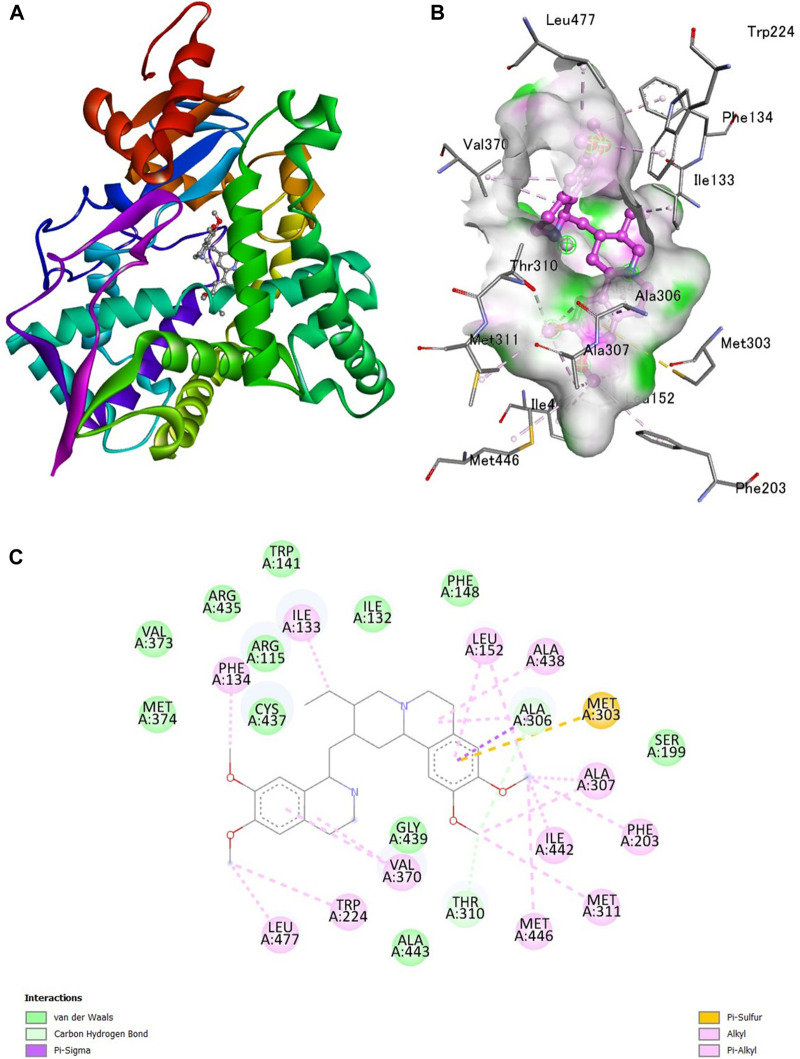
Interaction of emetine with aromatase receptor. **(A)** Protein ligand complex **(B)** 3D interaction of emetine with aromatase protein **(C)** 2D interaction of emetine with aromatase protein.

A docking study was done with specific pharmacological targets, such as the progesterone receptor and the aromatase enzymatic protein of breast cancer, which is implicated in the pathophysiology and induction of cancer, to explain the antioxidant activity of the produced compounds Figure 1.

**FIGURE 2:**
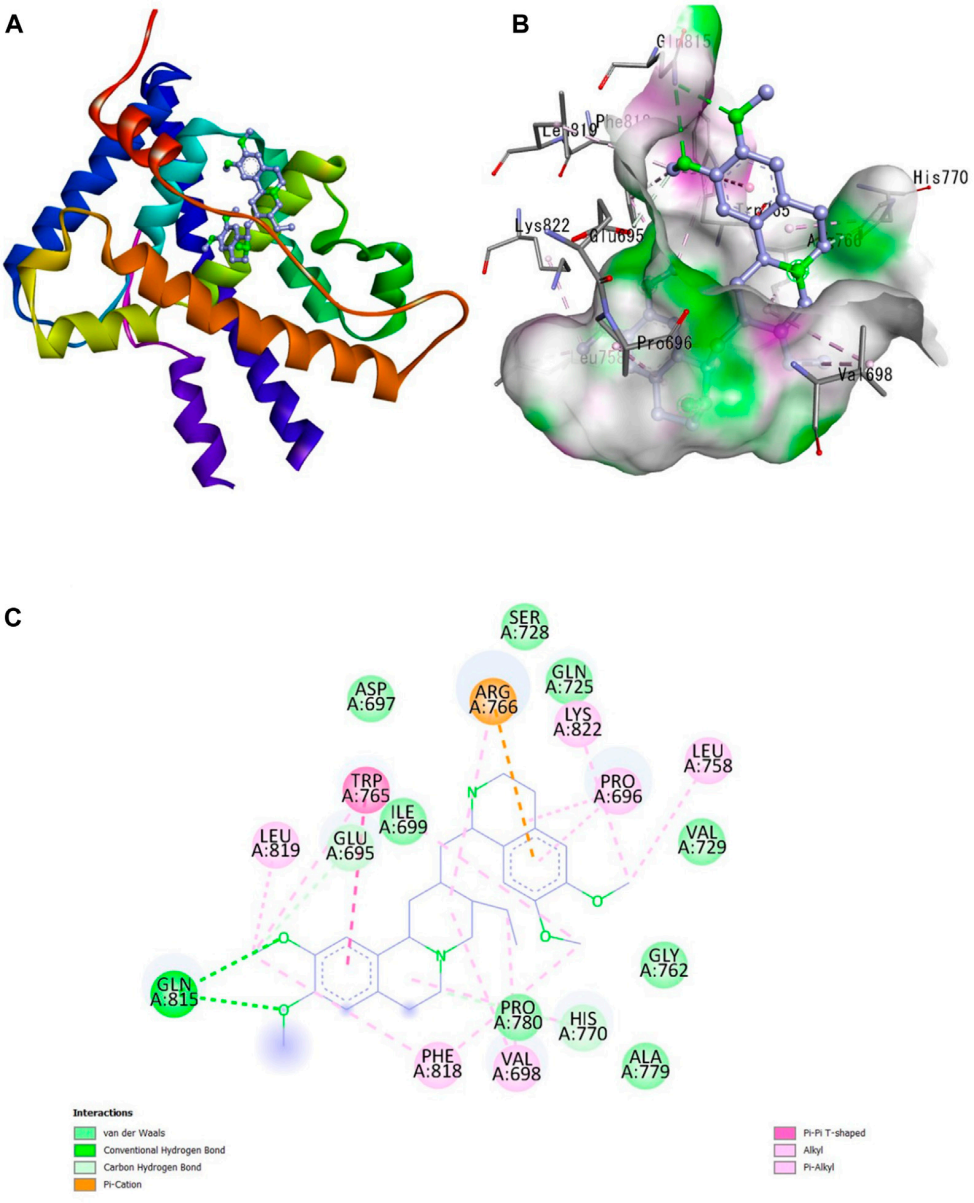
Interaction of emetine with progesterone receptor. **(A)** Protein ligand complex **(B)** 3D interaction of emetine with progesterone protein **(C)** 2D interaction of emetine with progesterone protein.

The Protein Data Bank was used to get the crystal structures of the Progesterone Receptor (PDB ID: 4OAR) and Aromatase (PDB ID: 3S7S), both of which had complexes with the reference medicines Ulipristal Acetate and Exemestane (EXM). The aromatase docking results showed that compounds 1, 2, and 3 have significant binding modes, with docking scores of −9.8 kcal/mol, −8.0 kcal/mol, and −7.7 kcal/mol, respectively. Compound 1 (Emetine) has a slightly lower dock score of −9.8 kcal/mol when compared to the control drug exemestane (−10.2 kcal/mol). Emetine, quercetin, and violanthin were the three phytochemicals with the highest binding affinities to PR, with respective values of −8.8 kcal/mol, −8.1 kcal/mol, and −7.8 kcal/mol. When compared to the control medication Ulipristal acetate (−8.1 kcal/mol) in these top three evaluations, Emetine was found to exhibit the best docking confirmation with high docking scores of −8.8 kcal/mol towards PR. Table 4 provides a summary of the H-bonds, binding affinities, and energy profiles of the chemicals toward the enzyme’s active site amino acids. The binding modes of compound 1 showed that they resembled the binding mode of the reference medication exemestane and fit more securely into the aromatase binding pocket by interacting with critical residues Leu152, Ile133, Phe134, Trp224, Met303, Ala 438, Ile442, Ala306, Thr310, Val370, Met446, and Leu477. Emetine forms two hydrogen bonds with Gln815 in the analysis of binding interactions to PR, which is identical to the binding configuration of the native ligand. In the binding pocket, the structure is also very effectively maintained by a number of hydrophobic interactions (Figure 2).

**TABLE 4:** The binding affinities in kcal/mol of particular drugs to aromatase and Progesterone Receptors, two cancer target sites.

Source	Phytochemicals	Structures	Docking scores against aromatase	Docking scores against progesterone receptor
Viola canescens	Emetine	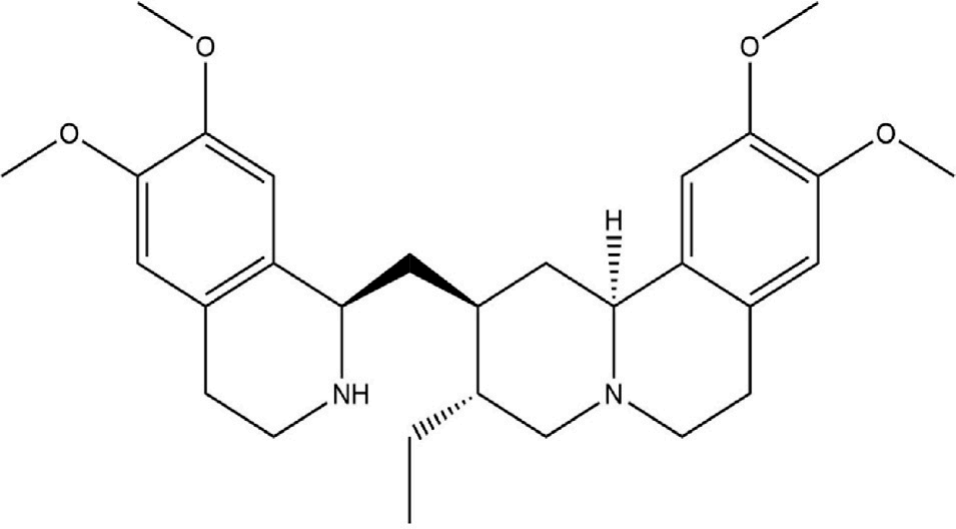	−9.8 kcal/mol	−8.8 kcal/mol
	Quercetin	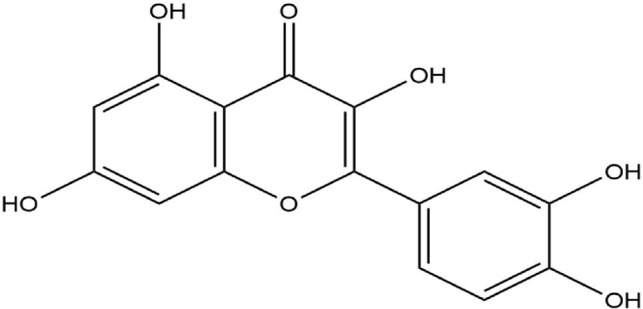	−8.0 kcal/mol	−8.1 kcal/mol
	Vioanthin	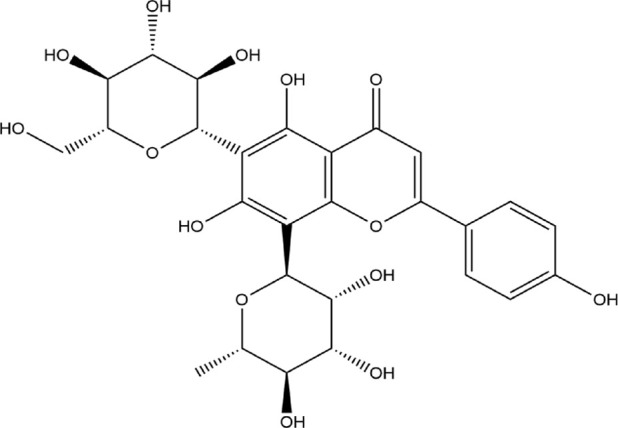	−7.7 kcal/mol	−7.6 kcal/mol
	Caffeic acid	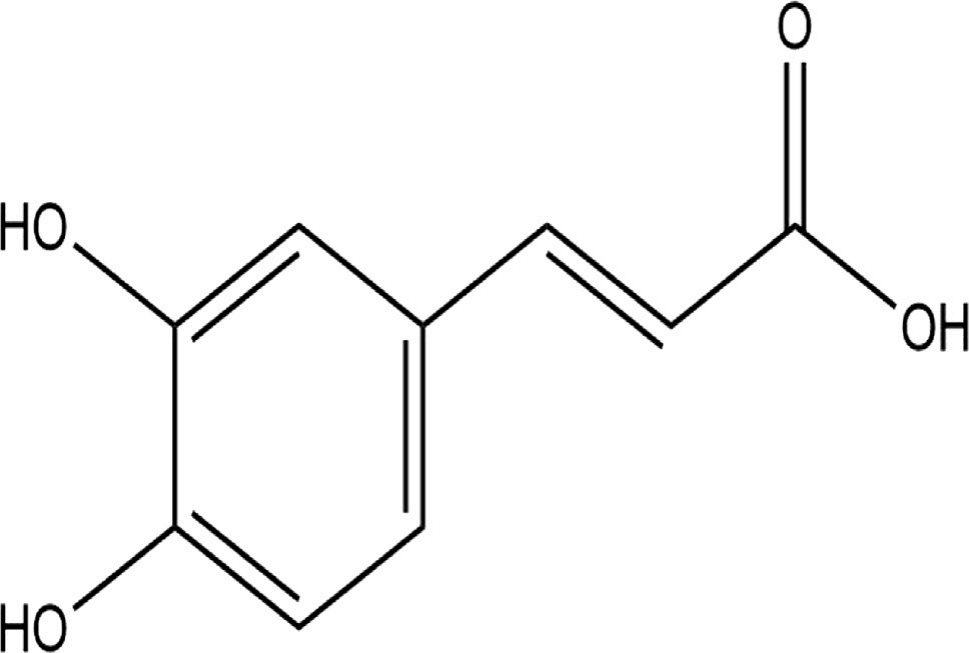	−6.9 kcal/mol	−5.9 kcal/mol
	Epicatechin	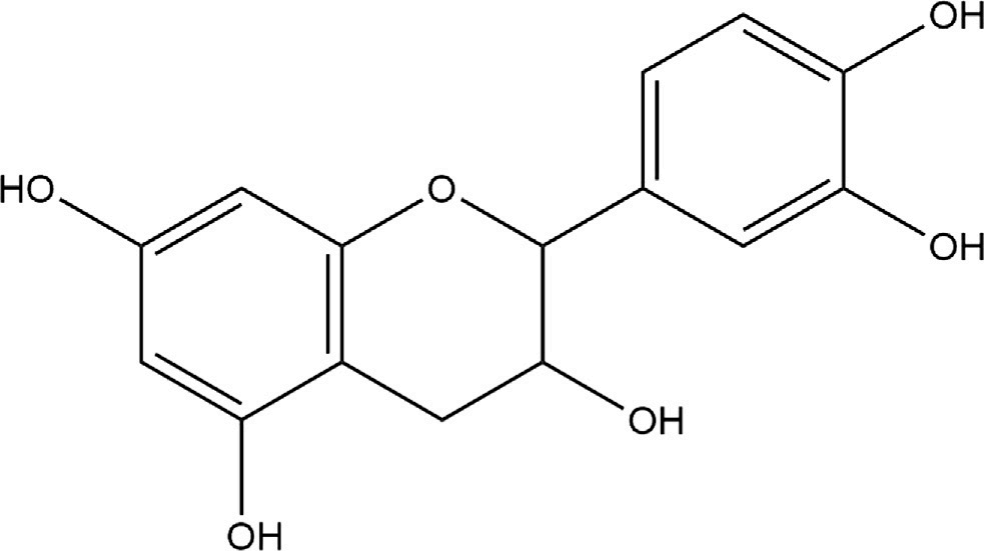	−7.0 kcal/mol	−6.6 kcal/mol
	Vanillic acid	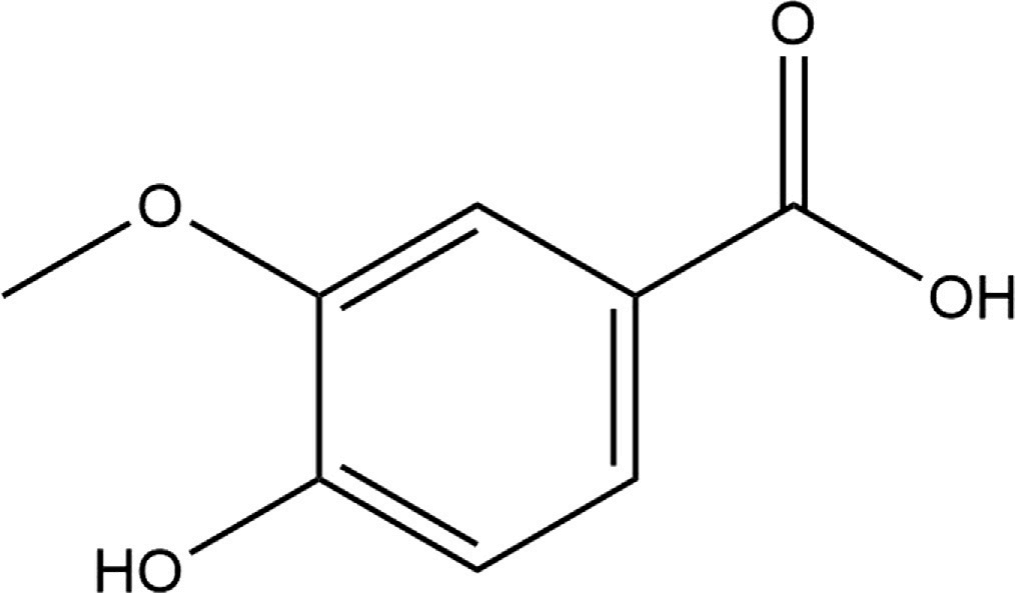	−6.1 kcal/mol	−5.5 kcal/mol
	p-coumaric acid	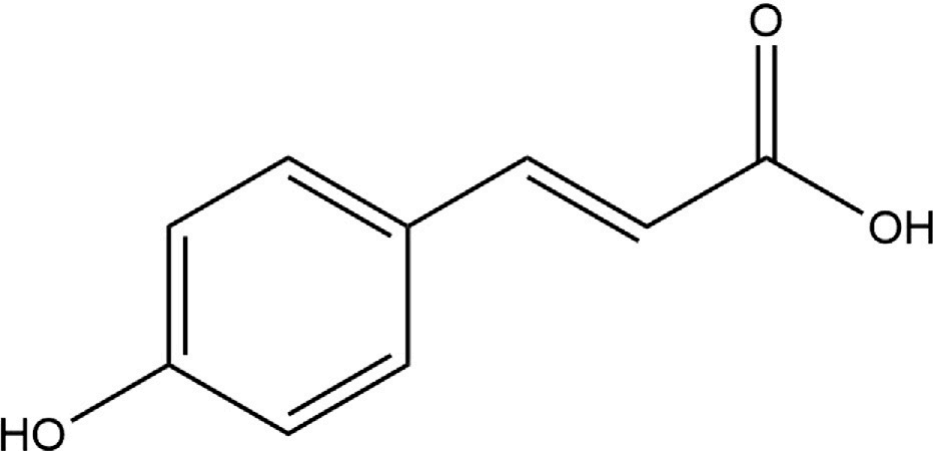	−6.7 kcal/mol	−5.7 kcal/mol
	Methyl salicylate	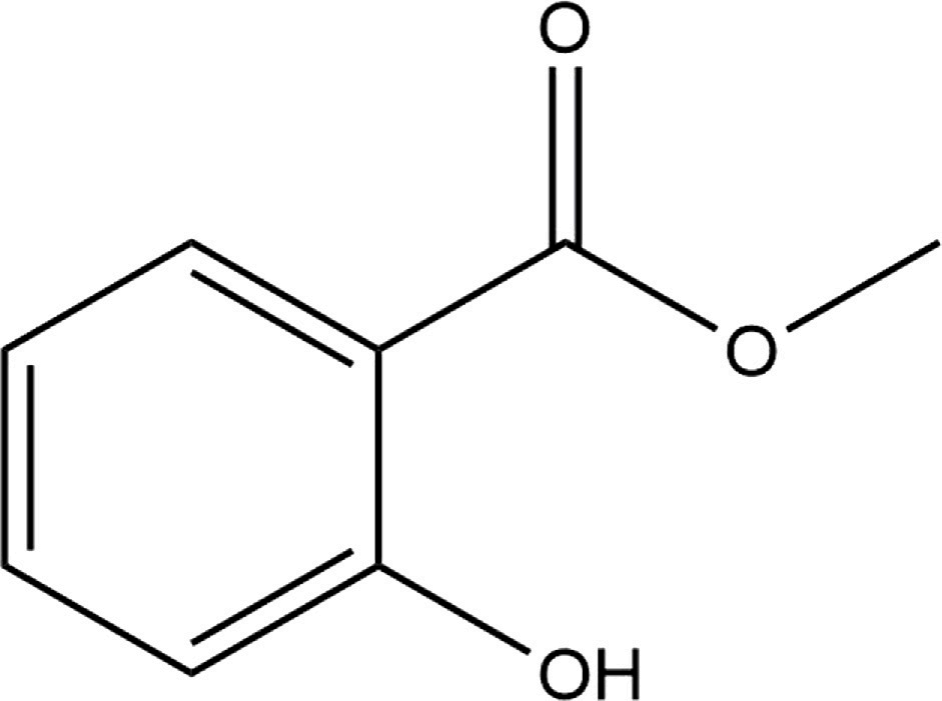	−5.4 kcal/mol	−5.1 kcal/mol
	Reference Drug Exemestane	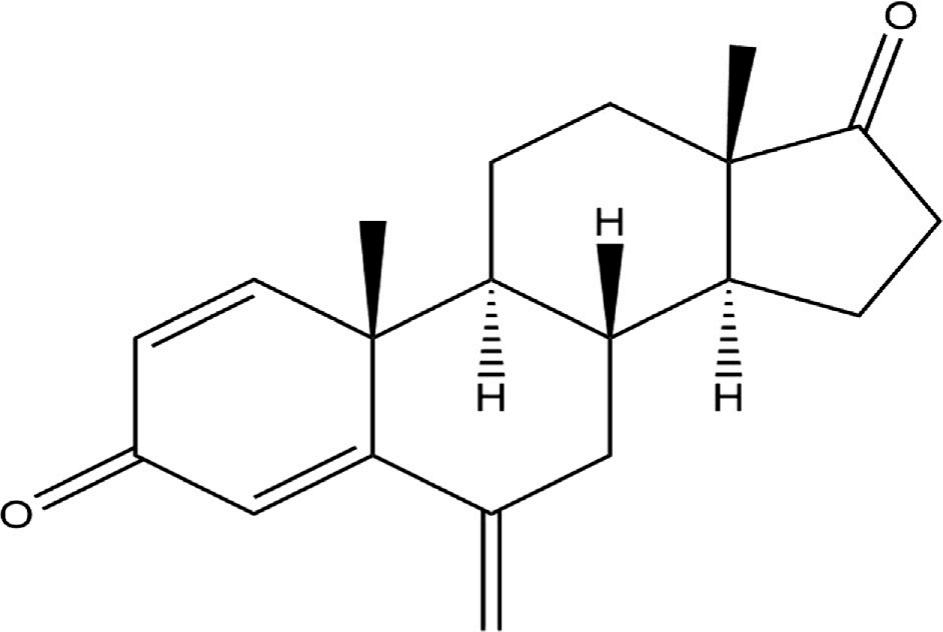	−10.4 kcal/mol	-
	Reference Drug Ulipristal acetate	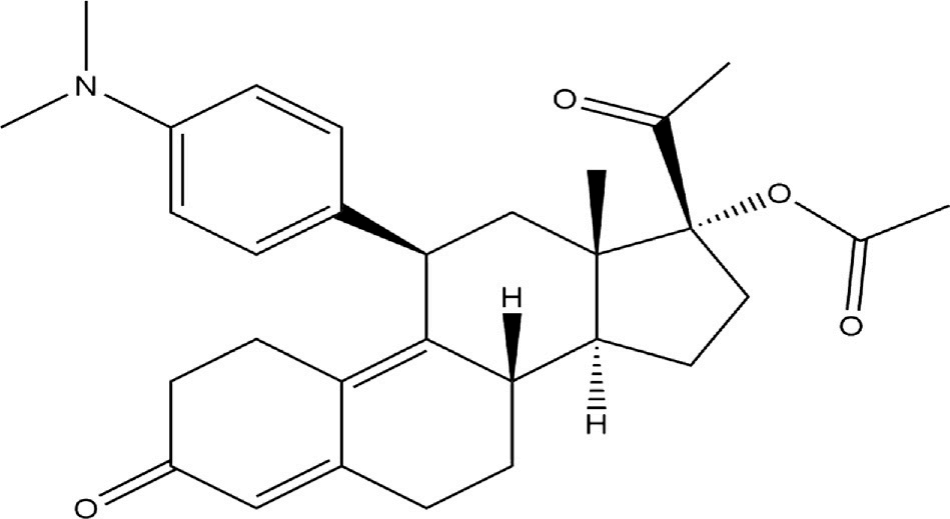	-	−8.1 kcal/mol

The considerable decline in aromatase and PR activity is therefore supported by these interactions.

Therefore, the results of the current study show that the synthetic chemicals will be the promising next-generation chemotherapeutic medications, which may be utilized to treat breast cancer and other conditions that are associated with it.

### 3.4 Pharmacokinetic and drug-likeness properties of phytocomponents

A chemoinformatic tool called Molinspiration was used to study three Phyto-molecules further in order to determine the physicochemical profile of the top hits for drug-likeness. In the table below, Lipinski’s rule of five is used to compute the bioactivity score for medications intended for oral administration. All MOL phytoconstituents had no violations of Lipinski’s rule of five, with the exception of violanthin (Lipinski’s violation = 2). The table displays the toxicity potential and drug-likeness of the MOL bioactive components. Results indicated that all components, with the exception of violanthin, are safe for usage and did not violate the Lipinski rule of five (Table 5).

**TABLE 5:** (a) A list of the phytochemicals that were chosen after applying Lipinski’s rule of five and information about their molinspiration bioactivity. (b) A compound’s molinspiration bioactivity score.

Source	A	compounds	milogP	TPSA	Number of atoms	Molecular weight	nON	nOHNH	Number of violation	Number of rotatable bonds	Volume
***Viola canescens***		Emetine	3.64	52.20	35	480.65	6	1	0	7	468.58
		Quercetin	1.68	131.35	22	302.24	7	5	0	1	240.08
		Violanthin	−1.09	250.96	41	578.52	14	10	3	4	478.10
	**b**	**Compounds**	**GPCR ligand**	**Ion channel modulator**	**Kinase inhibitor**	**Nuclear receptor ligand**		**Protease inhibitor**		**Enzyme inhibitor**	
		Emetine	0.22	0.10	−0.27	−0.20		0.08		−0.06	
		Quercetin	−0.06	−0.19	0.28	0.36		−0.25		0.28	
		Violanthin	0.05	−0.35	−0.02	−0.01		0.03		0.27	

### 3.5 Molecular dynamic simulation

The molecular interaction of the Emetine ligand with the target 4OAR receptor was examined using the i-Mode server. Using NMA, the docked complex of Emetine and 4OAR receptor was evaluated. The intricacy of the system’s internal coordinates was simulated using the i-Mode suite.

**FIGURE 3:**
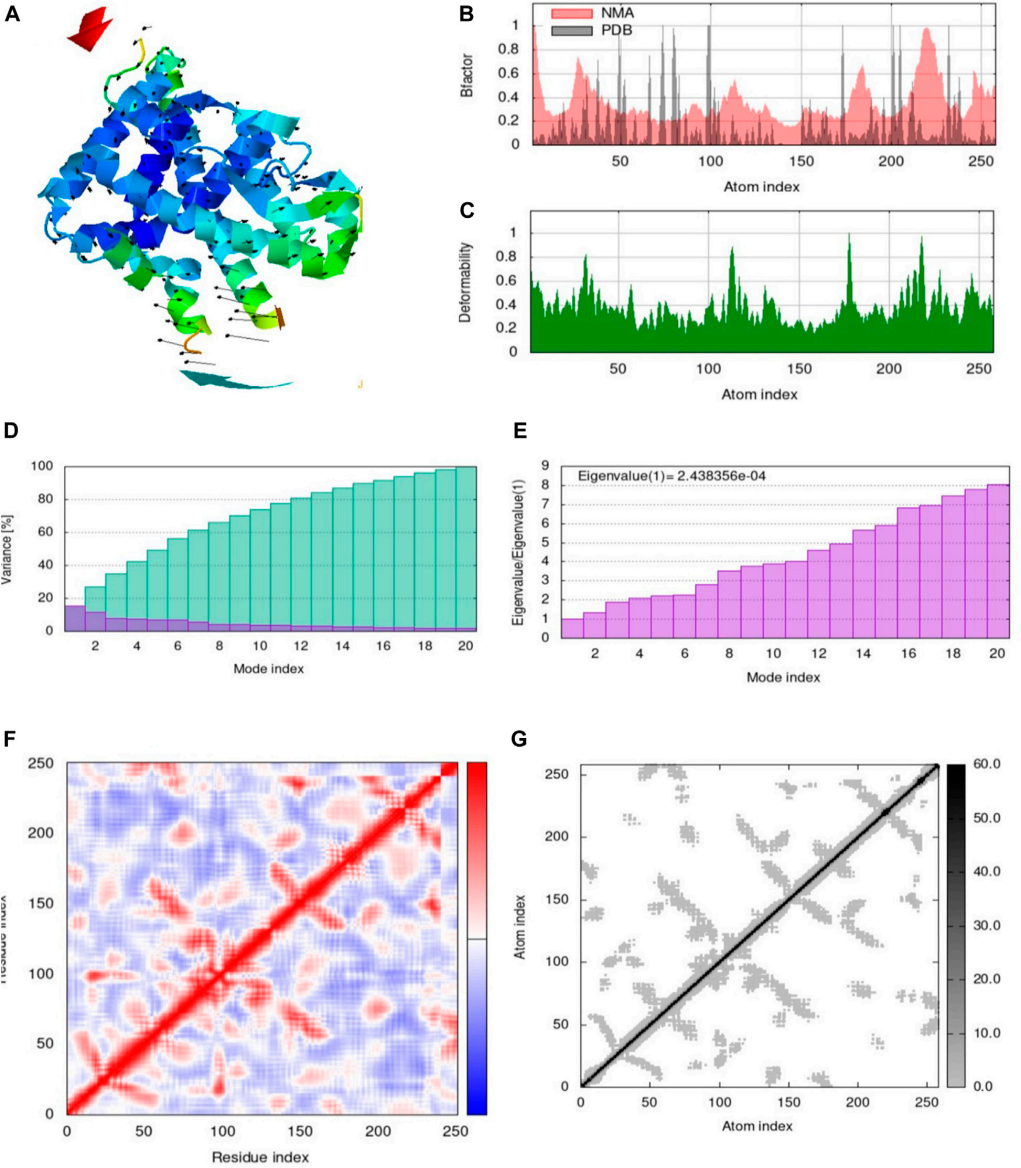
Ligand-protein complex molecular dynamics simulation, showing **(A)** spin prediction of the ligand–receptor interaction; **(B)** deformability; **(C)** B-factor; **(D)** eigenvalue; **(E)** Variance; **(F)** covariance matrix depicting the coupling between pairs of residues (red), uncorrelated (white), or anti-correlated (blue) motions; **(G)** elastic network analysis defining.

The system’s trajectory was examined in order to ascertain the deformability. Figure 3B displays the spin prediction of the ligand-receptor interaction and other data from the molecular dynamics’ simulation of the Emetine-4OAR complex. The results of the complex trajectory showed that the coordinates from 0 to 1A were slightly deformed. This shows a stable interaction between the ligand and very little distortion (Figure 3). NMR measurements of the 4OAR receptor and ligand system trajectory revealed traces of certain atomic oscillations. The calculated eigen score of 2.438,356 × 10^−04^ is displayed in Figure 3E. The Emetine-4OAR complex atomic pairs were also uncovered using covariance matrix analysis. Correlated segments were shown in red, uncorrelated segments in blue, and uncorrelated segments in white in the analysis. Figure 3A depicts the modification of the 4OAR binding groove as well as the integration of the 4OAR protein residues with the ligand. Using distance-based spring analysis, the model’s elastic network revealed pairs of atomic coordinates. In the network plot, each dot stands in for a spring and is colored according to how stiff the complex is in proportion to the matching atomic pairs. The degree of compactness and stability of the binding complex system is represented by grey-colored spring models (see Figure 3G). These significant findings highlight the receptor’s complex stiffness and steady binding, which are accompanied by certain atomic variations and have a low deformation index.

## 4 Discussion

Free radicals produced in the human body due to various metabolic activities can cause minor to severe health issues. Cancer is a severe medical condition triggered by excessive accumulation of free radicals (oxidative stress) (Ndhlala et al., 2010). However, most cell types of the human body minimize the adverse effects of oxidative stress through their built-in antioxidant mechanisms; in most cases, these built-in antioxidant mechanisms alone are insufficient to eliminate excessive free radicals effectively (Liao et al., 2008). Numerous plant-derived substances are known for their potential to improve the antioxidant abilities of cells, thus reducing the chances of cancer and other health issues related to oxidative stress. *V. canescens* is used in traditional healthcare systems for various ailments, including cancer. Therefore, the current study aimed to confirm its traditional uses by determining its ability to scavenge free radicals, minimizing oxidative stress.

In the assay, the total antioxidant potential of plant-derived material is assessed by their abilities to convert/reduce tetraoxomolybdate (IV) to pentaoxomolybdate (VI). A number of flavonoids and other polyphenols are reported for their reducing potential (Singh et al., 2014). The maximum potentials of polar solvent fractions, CTA and CTF, could be attributed to the presence of polyphenols in these fractions.

DPPH radical scavenging assay is an important and economical assay to assess the radical scavenging abilities of plant-derived materials. The assay measures the antioxidant potential of extracts/fractions by reducing DPPH, i.e., converting DPPH to DPPH-H. The reduction of DPPH to DPPH-H is indicated by a decrease in absorbance at 517 nm (Tejero et al., 2014). Results of the current study revealed the significant DPPH scavenging abilities of used extracts/fractions, which could be associated with the hydrogen donating abilities of phytochemicals present in these extracts/fractions. The maximum activities of crude alkaloids and crude flavonoids suggest their highest DPPH-reducing potential.

Emetine, quercetin, and violanthin isolated from *V. canescens* (Chandra et al., 2015) are most likely responsible for the antioxidant potential of the plant. The three isolated compounds were subjected to docking analysis to better understand the observed antioxidant properties of *V. canescens.*

In particular, computer-assisted medication design has a substantial impact on promoting pharmaceutical development (Khan et al., 2020). Silico approaches are the most influential computational tools for analyzing structural molecular ligand-receptor interactions and providing a new understanding of suitable biochemical pathways for natural chemicals (Khan et al., 2019). The *in silico* molecular method can also illuminate potential targets and mechanisms underlying various pharmacological actions. The molecular modeling study was carried out to clarify the molecular pathways further better to target the outcomes of the current experimental findings. The docking technique used in this study, in particular, gave useful insight into the biologically active isolates’ bindings to various protein targets at the molecular and cellular levels. These protein targets, such as anti-oxidant, anti-depressant, and anti-diarrheal cascades, are essential in pharmacological pathways. Additionally, it provides more information regarding potential mechanisms of action and binding styles within the binding pocket of enzymes (Chy et al., 2020).

Compounds were docked against the progesterone receptor (PR) and the aromatase protein, two target proteins. The ligand-receptor complex interacts strongly and favorably, as evidenced by negative and low binding energies (Adnan et al., 2020). According to these findings, Emetine showed a promising docking score compared to Ulipristal Acetate and Exemestane, the reference medicines, among the three isolates with anti-oxidant activity.

The online prediction tool Molinspiration was used to conduct additional research on all compounds. It assessed drug-likeness and other properties using Lipinski’s rule of five (Adnan et al., 2019), compounds with a molecular weight of 500 amu or less, 10 hydrogen bond acceptors, five hydrogen bond donors, and favorable features for absorption and bioavailability (Daina et al., 2014).

## Data Availability

The original contributions presented in the study are included in the article/supplementary material, further inquiries can be directed to the corresponding authors.

## References

[B1] Adnan M. Chy M. N. U. Kamal A. Chowdhury M. R. Islam M. S. Hossain M. A. (2020). Unveiling pharmacological responses and potential targets insights of identified bioactive constituents of cuscuta reflexa roxb. Leaves through *in vivo* and *in silico* approaches. Leaves through Vivo Silico Approaches. Pharm. (Basel) 13, 50. 10.3390/ph13030050 PMC7151675 32245131

[B2] Adnan M. Nazim Uddin Chy M. Mostafa Kamal A. T. M. Azad M. O. K. Paul A. Uddin S. B. (2019). Investigation of the biological activities and characterization of bioactive constituents of ophiorrhiza rugosa var. prostrata (D.don) and mondal leaves through *in vivo*, *in vitro*, and *in silico* approaches. Molecules 24, 1367. 10.3390/molecules24071367 30965575 PMC6480688

[B3] Ahmad I. Ibrar M. Barkatullah and Ali N. (2011). Ethnobotanical study of tehsil kabal, Swat District, KPK, Pakistan. J. Bot. 2011, 1–9. 10.1155/2011/368572

[B4] Chandra D. Kohli G. Prasad K. Bisht G. Punetha V. D. Khetwal K. S. (2015). Phytochemical and ethnomedicinal uses of family Violaceae. Curr. Res. Chem. 7, 44–52. 10.3923/crc.2015.44.52

[B5] Chy M. N. U. Adnan M. Rauniyar A. K. Amin M. M. Majumder M. Islam M. S. (2020). Evaluation of anti-nociceptive and anti-inflammatory activities of Piper sylvaticum (Roxb.) stem by experimental and computational approaches. Adv. Traditional Med. 20, 327–341. 10.1007/s13596-019-00395-9

[B6] Cox-Georgian D. Ramadoss N. Dona C. Basu C. (2019). “Therapeutic and medicinal uses of terpenes,” in Medicinal plants: from farm to pharmacy. Editors JOSHEE N. DHEKNEY S. A. PARAJULI P. (Berlin, Germany: Springer International Publishing).

[B7] Daina A. Michielin O. Zoete V. (2014). iLOGP: a simple, robust, and efficient description of n-octanol/water partition coefficient for drug design using the GB/SA approach. J. Chem. Inf. Model 54, 3284–3301. 10.1021/ci500467k 25382374

[B8] De Torre M. P. Cavero R. Y. Calvo M. I. Vizmanos J. L. (2019). A simple and a reliable method to quantify antioxidant activity in viv Antioxidants (Basel) 8 (5), 142. 10.3390/antiox8050142 31121854 PMC6562907

[B9] Göçer H. Gülçin I. (2011). Caffeic acid phenethyl ester (CAPE): correlation of structure and antioxidant properties. Int. J. Food Sci. Nutr. 62, 821–825. 10.3109/09637486.2011.585963 21631390

[B10] Huang D. Ou B. Prior R. L. (2005). The chemistry behind antioxidant capacity assays. J. Agric. Food Chem. 53, 1841–1856. 10.1021/jf030723c 15769103

[B11] Kalita P. Tapan B. K. Pal T. K. Kalita R. (2013). Estimation of total flavonoids content (TFC) and anti oxidant activities of methanolic whole plant extract of Biophytum sensitivum Linn. J. Drug Deliv. Ther. 3, 33–37. 10.22270/jddt.v3i4.546

[B12] Kedare S. B. Singh R. P. (2011). Genesis and development of DPPH method of antioxidant assay. J. Food Sci. Technol. 48, 412–422. 10.1007/s13197-011-0251-1 23572765 PMC3551182

[B13] Khan M. F. Kader F. B. Arman M. Ahmed S. Lyzu C. Sakib S. A. (2020). Pharmacological insights and prediction of lead bioactive isolates of Dita bark through experimental and computer-aided mechanism. Biomed. Pharmacother. 131, 110774. 10.1016/j.biopha.2020.110774 33152933

[B14] Khan S. Nazir M. Raiz N. Saleem M. Zengin G. Fazal G. (2019). Phytochemical profiling, *in vitro* biological properties and *in silico* studies on Caragana ambigua stocks (Fabaceae): a comprehensive approach. Industrial Crops Prod. 131, 117–124. 10.1016/j.indcrop.2019.01.044

[B15] Liao H. Banbury L. K. Leach D. N. (2008). Antioxidant activity of 45 Chinese herbs and the relationship with their TCM characteristics. Evid. Based Complement. Altern. Med. 5, 429–434. 10.1093/ecam/nem054 PMC2586310 18955214

[B16] López-Blanco J. R. Aliaga J. I. Quintana-Ortí E. S. Chacón P. (2014). iMODS: internal coordinates normal mode analysis server. Nucleic Acids Res. 42, W271–W276. 10.1093/nar/gku339 24771341 PMC4086069

[B17] Mendelsohn L. D. (2004). ChemDraw 8 ultra, windows and macintosh versions. J. Chem. Inf. Comput. Sci. 44 (6), 2225–2226. 10.1021/ci040123t

[B18] Muhammad A. Dirk H. (2010). Medicinal plant abundance in degraded and reforested sites in northwest Pakistan. Mt. Res. Dev. 30, 25–32. 10.1659/mrd-journal-d-09-00017.1

[B19] Ndhlala A. R. Moyo M. Van Staden J. (2010). Natural antioxidants: fascinating or mythical biomolecules? Molecules 15, 6905–6930. 10.3390/molecules15106905 20938402 PMC6259562

[B20] Prieto P. Pineda M. Aguilar M. (1999). Spectrophotometric quantitation of antioxidant capacity through the formation of a phosphomolybdenum complex: specific application to the determination of vitamin E. Anal. Biochem. 269, 337–341. 10.1006/abio.1999.4019 10222007

[B21] Qasier M. Omer S. (1985). Violaceae. *Flora of Pakistan* .Nasir Ali, S.I., E

[B22] Saeed N. Khan M. R. Shabbir M. (2012). Antioxidant activity, total phenolic and total flavonoid contents of whole plant extracts Torilis leptophylla L. BMC Complement. Altern. Med. 12, 221. 10.1186/1472-6882-12-221 23153304 PMC3524761

[B23] Sajjadi M. Karimi E. Oskoueian E. Iranshahi M. Neamati A. (2019). Galbanic acid: induced antiproliferation in estrogen receptor-negative breast cancer cells and enhanced cellular redox state in the human dermal fibroblasts. J. Biochem. Mol. Toxicol. 33, e22402. 10.1002/jbt.22402 31576639

[B24] Singh M. Jha A. Kumar A. Hettiarachchy N. Rai A. K. Sharma D. (2014). Influence of the solvents on the extraction of major phenolic compounds (punicalagin, ellagic acid and gallic acid) and their antioxidant activities in pomegranate aril. J. Food Sci. Technol. 51, 2070–2077. 10.1007/s13197-014-1267-0 25190865 PMC4152477

[B25] Tavassoli S. Djomeh Z. E. (2011). Total phenols, antioxidant potential and antimicrobial activity of methanol extract of rosemary (Rosmarinus officinalis L.). Glob. Veterinaria 7, 337–341.

[B26] Tejero J. S. Gayoso S. Caro I. Cordoba-Diaz D. N. Mateo J. Basterrechea J. E. (2014). Comparative analysis of the antioxidant and free-radical scavenging activities of different water-soluble extracts of green, black and oolong tea samples. Food Nutr. Sci. 05 (22), 2157–2166. 10.4236/fns.2014.522228

[B27] Trott O. Olson A. J. (2010). AutoDock Vina: improving the speed and accuracy of docking with a new scoring function, efficient optimization, and multithreading. J. Comput. Chem. 31, 455–461. 10.1002/jcc.21334 19499576 PMC3041641

[B28] Zengin G. Cakmak Y. S. Guler G. O. Aktumsek A. (2010). *In vitro* antioxidant capacities and fatty acid compositions of three Centaurea species collected from Central Anatolia region of Turkey. Food Chem. Toxicol. 48, 2638–2641. 10.1016/j.fct.2010.06.033 20600531

